# Large-scale photonic integrated circuits for advanced displays

**DOI:** 10.1093/nsr/nwag234

**Published:** 2026-04-21

**Authors:** Zhujun Shi, Barry Silverstein

**Affiliations:** Department of Physics and Astronomy, University of Pittsburgh, USA; Center for eXtended Reality (CXR), University of Rochester, USA

## Abstract

Photonic integrated circuits are opening new paths to compact, efficient, and highly functional display systems for next-generation human-computer interaction.

Display technology has long been a major driver of optics and photonics innovation, and it now faces a pivotal transition. Next-generation display platforms [1], such as augmented reality (AR), virtual reality, and holographic displays, aim to deliver immersive and interactive visual experiences on wearable platforms, transforming how we interact with digital content. Achieving this vision, however, demands performance metrics that push well beyond the limits of current display architectures and require fundamentally new ways to generate, manipulate, and deliver light.

Compared with today’s displays, next-generation display systems must improve in many areas including power efficiency, precise light control, and extreme miniaturization. First, power efficiency becomes increasingly critical as immersive displays require ever-higher pixel densities, making heat management and pixel aperture ratio losses challenging as the native efficiency of light-emitting devices degrades under both conditions [2]. Second, future displays require precise control over light properties beyond simple intensity modulation. They need versatile control over the light’s phase and amplitude to provide 3D depth cues, and careful tailoring of the spectral, polarization, and coherence properties of light to improve uniformity and reduce eye glow. Finally, aggressive miniaturization is needed, especially for AR displays as all-day wearables must be ultralight and compact, and socially acceptable necessitating a paradigm shift away from traditional, bulk-optic-based display architectures.

Integrated photonics with coherent laser sources offers a powerful toolkit to address these challenges. Photonic integrated circuits (PICs) provide precise, programmable control over optical phase, amplitude, spectrum, and polarization within a compact platform. A substantial efficiency gain arises from shaping the emitted light so that it enters the display system already in the form it requires, carrying the correct etendue (spatial and angle), spectral, and polarization distributions, thereby eliminating the cascade of lossy filters, diffusers, and bulky relay optics in conventional optical architectures. The rapid progress of visible-wavelength photonics, originally accelerated by bioimaging and quantum technologies, has made it an increasingly appealing platform for display applications, which operate across the red, green, and blue (RGB) spectrums. Combined with complementary metal-oxide-semiconductor (CMOS) compatibility, heterogeneous laser integration, and emerging active on-chip control, PICs now offer a scalable route for dense integration of sources, modulators, and emitters. This translates into new capabilities for display systems: bespoke illumination, compact beam-steering, programmable holography, and entirely new classes of display architectures.

In the near term, the most immediate impact of PICs comes from their integration into existing display architectures such as liquid crystal display, LCoS (liquid-crystal-on-silicon) [3], and micro-electro-mechanical system displays, primarily through source integration and illumination engineering. The first step in generating a color image is to combine the red, green, and blue light sources either into a singular source or spatially aggregated. In some cases, especially as the display resolution or field of view increases, more than one source per color may be required to improve image quality or resolution. Today’s color-mixing modules rely on free-space optics such as cascaded dichroic mirrors, which are bulky, inefficient, and scale poorly as the number of sources increases. PIC-based color mixing, by contrast, uses wavelength-selective routing and multiplexer/demultiplexer networks to combine multiple lasers on a single chip with high efficiency in a compact and scalable format, and recent demonstrations have shown strong performance as these approaches move toward commercialization [4] (Fig. 1a). Illumination engineering is another area where PICs offer distinct advantages: they can consolidate functions such as beam expansion, polarization control, and light extraction into a single chip while simultaneously providing precise control over the illumination wavefront, including its spatial, angular, spectral, and polarization distribution, to match the resolution, field of view, and optical constraints of the display system. These capabilities align naturally with the emerging field of on-chip wavefront shaping, in which integrated photonic devices, often combined with metasurfaces, sculpt the emitted light field directly from the chip [5]. Recent work has demonstrated multifunctional and multiwavelength wavefront control by patterning nanopillars on slab waveguides or on arrays of single-mode emitters. Furthermore, dynamic illumination control becomes possible when active modulators are integrated. This enables zonal illumination architectures, in which the display is illuminated only in the regions currently contributing to the final image—reducing power consumption, enabling efficient uniformity correction, and improving display contrast. Recent system-level demonstrations [6] have shown that these concepts can be realized in practice. Large-aperture visible-light PICs containing over a thousand waveguides and ten thousand emitters have been integrated with LCoS displays, achieving >80% volume reduction and a 5× improvement in efficiency while delivering panel-scale illumination with high brightness, contrast, and uniformity (Fig. 1b).

**Figure 1.**
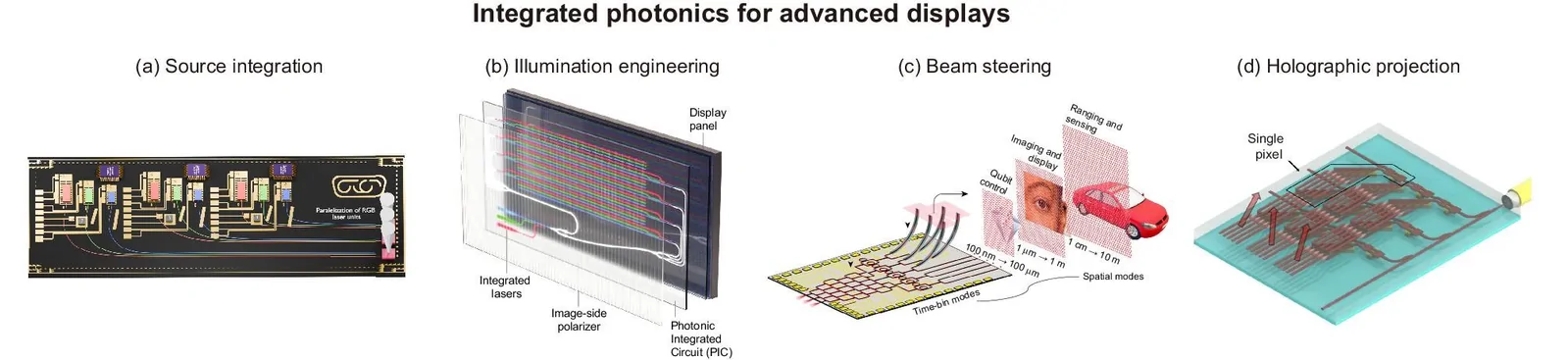
Representative examples of PIC-enabled display functions, including (a) source integration for compact light engines [4], (b) illumination engineering for panel-based displays [6], (c) chip-scale beam steering [8], and (d) holographic wavefront generation [10]. Figures reprinted with permission from [4], BrillianceRGB; [6] and [8], Springer Nature; and [10], SPIE.

Looking further ahead, PICs open the door to entirely new display architectures in which the photonic chip itself becomes the primary image-forming engine, integrating light generation, wavefront shaping, and pixel-level modulation on a single platform. Key enabling elements include integrated modulators [7], PIC-based micromechanical cantilevers [8], and on-chip source modulation [9], corresponding, respectively, to image formation through direct amplitude/phase modulation, mechanical beam scanning, and wavelength-based (spectral) scanning. These mechanisms can operate independently or in hybrid configurations. Representative examples of chip-scale beam steering and holographic wavefront generation are shown in Fig. 1c and d. For example, a full 2D image can be produced by pairing 1D mechanical scanning with 1D on-chip switching using Mach–Zehnder interferometer trees, or by combining 1D switching with 1D wavelength-steered beam scanning. Such systems can offer pixel plane location adjustment to correct for vergence–accommodation mismatch through multiple methods of phase adjustment. Alternatively, a long-standing goal in the field is a true holographic display capable of generating full 3D wavefronts, thereby addressing the vergence–accommodation conflict holistically [10]. Achieving holography requires pixel-level control of both amplitude and phase over a large optical aperture, which is commonly referred to as optical phased arrays (OPAs) [11]. Although OPAs have seen significant progress in light detection and ranging (LiDAR), display systems impose fundamentally different and more stringent requirements: arcminute-level angular resolution to match human visual acuity, high beam quality with minimal sidelobes, large effective apertures, multiwavelength operation across RGB, and total power budgets on the order less than a few hundred milliwatts. For direct-view AR, the entire device must also be highly transparent so that real-world light passes through without distortion or unwanted diffraction. These systems also must minimize the display leakage to the outside world to manage privacy and social acceptance. Such light can be more easily managed by using tightly controlled light with metasurfaces or filters that are carefully tuned. Collectively, these constraints push integrated photonics into a new design space, one that demands ultradense visible-wavelength emitter arrays with precise amplitude and phase control, low-loss RGB operation, and fabrication on transparent substrates.

Integrated photonics for displays is an emerging application, and many concepts remain in early research stages. Substantial technical and manufacturing challenges must be resolved before these technologies can support scalable, consumer-grade products. Across architectures, several bottlenecks consistently dominate. First, developing high-performance modulators for the visibility remains a key challenge. Compared with telecom photonics, display applications place tighter demands on integration density and power consumption, while still requiring high stability and good repeatability. Second, refining robust and scalable laser–PIC integration: visible lasers span multiple III–V materials, require tight alignment tolerances, and must be packaged on transparent, thermally constrained substrates within millimeter-scale volumes. Third, creating a robust foundry ecosystem for visible photonics, including standardized materials platforms, process design kits (PDKs), and high-yield processes is essential to meet the tighter tolerances and new substrate requirements imposed by visible-light operation. Another important requirement is tight cointegration with CMOS driver electronics and design workflows that link photonic devices, electronic backplanes, and system-level control, since display performance and scalability depend on the full optoelectronic stack rather than the photonic chip alone. Encouragingly, advanced displays share many key requirements with other important domains such as biophotonics and quantum technologies, which also require low-loss visible waveguides, efficient laser integration, compact modulators, and scalable photonics–electronics co-packaging. This cross-domain synergy is enabling a rapidly improving manufacturable visible PIC platform that will allow integrated photonics to become available for next-generation displays.
